# Conditional Relative Odds Ratio and Comparison of Accuracy of Diagnostic Tests Based on 2×2 Tables

**DOI:** 10.2188/jea.16.145

**Published:** 2006-07-13

**Authors:** Sadao Suzuki

**Affiliations:** 1 Department of Health Promotion and Preventive Medicine, Nagoya City University Graduate School of Medical Sciences.

**Keywords:** Diagnosis, Sensitivity and Specificity, Odds Ratio, Meta-Analysis

## Abstract

In order to evaluate the accuracy of diagnostic tests based on 2×2 tables, a number of indices were used, some of which are occasionally used inappropriately. This paper demonstrates the characteristics and problems with those indices, and introduces several methods to compare the accuracy of two diagnostic tests. The author summarizes existing indices based on 2×2 tables, agreement rate, kappa (*κ*), and odds ratio, and reviews their characteristics to find better indices by which to compare two diagnostic tests using hypothetical examples. Because only the odds ratio is not affected by prevalence, the relative odds ratio is the most appropriate index for comparing diagnostic accuracy. In order to decrease selection bias, giving the two tests to the same individuals is preferred. However, no standard method has been established to obtain the standard error of relative odds ratios. In this case, using the newly proposed conditional relative odds ratio (*CROR*), based on McNemar’s odds ratio, the standard error is available. The *CROR* is a less biased index when the two tests were given to the same individuals, and it is also preferable in light of its ethical and economic advantages. However, a large base population is required for the two tests to be highly accurate and produce few discordant results.

## INTRODUCTION

Diagnostic accuracy is commonly measured by sensitivity and specificity of which trade-off relationship can be presented in the form of a receiver-operating characteristic (ROC) curve. One summary index of diagnostic test accuracy is based on the area under the ROC curve^1^^-^^3^ representing for an integrated discriminative ability of a diagnostic test over cut-off points. Others are based on a single 2×2 table of a specific cut-off point.^4^^-^^9^ In this paper, the author first selects several summary statistics belonging to the latter category, then focuses on the comparison of diagnostic accuracy using the odds ratio.^9^^,^^10^^-^^12^ Lastly, ways to summarize diagnostic accuracy using the meta-analytic method are introduced.

## METHODS

As described in Table 1, diagnostic test accuracy based on a 2×2 table is most commonly presented by two trade-off indices, sensitivity=*θ*=*a*/D, and specificity=*ϕ*=*d*/ND. When we need to describe diagnostic accuracy by a single index, there are at least three options, *i.e.*, agreement rate (*AR*), kappa^4^ (*κ*), and odds ratio (*OR*). The author presents these statistics along with their strengths and weaknesses, and then focuses on the odds ratio to compare the two diagnostic tests administered to the different or same subjects. In the last approach, two meta-analytic methods for a comparison of diagnostic accuracy are reviewed. For each approach, the author provides a hypothetical example to show the actual computational steps. All analyses were re-performed using the SAS release 8.2 (SAS Institute Inc., Cary, NC).^13^ The code is presented in the appendix.

**Table 1.** Indices of diagnostic accuracy based on 2×2 tables.

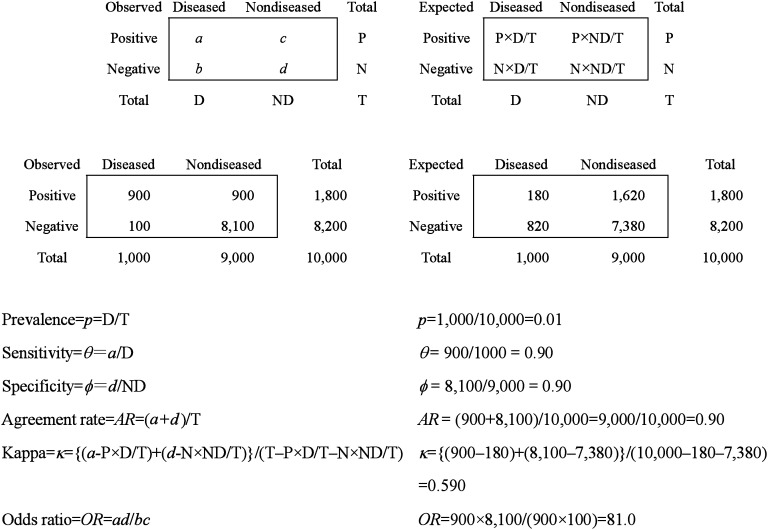

## APPROACHES

### 1. Approach to Evaluation of Diagnostic Test Accuracy

One of the widely used indices for diagnostic accuracy is *AR*, alternatively percent agreement. It is calculated as the number of correctly categorized subjects over the total number; *AR*=(*a*+*d*)/T in Table 1. *AR* is computationally simple and intuitively interpretable. It could be manipulated to *pθ*+ (1−*p*)*ϕ*, and this is interpreted as the weighted mean of sensitivity and specificity by prevalence.

Among several statistics^5^ proposed for 2×2 table data to improve *AR* with regard to removing chance agreement, *κ*^4^ has frequently received high marks. As shown in Table 1, the index is calculated by subtracting the expected number of correctly diagnosed individuals from both the numerator and denominator of *AR*. Prevalence remains in the formula as follows:

$$\kappa =\frac{a+d-\{\text{expected}(a)+\text{expected}(d)\}}{T-\{\text{expected}(a)+\text{expected}(d)\}}=\frac{a+d-(P\times D+N\times ND)/T}{T-(P\times D+N\times ND)/T}=\frac{2p(1-p)(\theta +\phi -1)}{2p(1-p)(\theta +\phi 1)+\{1-p\theta (1-p)\phi \}}$$

The *OR*, frequently used in causality studies, is also used to evaluate diagnostic accuracy.^7^^-^^9^ In causality studies, the *OR*s stand for the strength of the relationship between exposure and disease. This is easily interpreted as the relationship between test results and the presence of the disease. The *OR* is also interpreted as the ratio of true-positive to false-positive odds. The index is manipulated to 1/{(1/*θ*−1)(1/*ϕ*−1)}, in which prevalence is cancelled out.

### 2. Approach to Comparison of Diagnostic Test Accuracy

Among the above three indices for the evaluation of diagnostic test accuracy, only the *OR* is not affected by prevalence, which is a valuable feature when comparing accuracy. In this section, the author demonstrates how to compare the diagnostic accuracy of two tests using the *OR* among both different and the same subjects.

#### 2-1. Comparison of Diagnostic Accuracy of Two Tests Given to Different Subjects Indices Based on 2×2 Tables by Test

When we compare the diagnostic accuracy of two tests, X and Y, applied to different subjects, relative odds ratio (*ROR*), the ratio of the two *OR*s, is available.^9^ As shown in Table 2, the index is calculated as follows:

$$ROR=\frac{{OR}_{\text{X}}}{{OR}_{\text{Y}}}=\frac{ad/bc}{a^{\prime }d^{\prime }/b^{\prime }c^{\prime }}=\frac{adb^{\prime }c^{\prime }}{bca^{\prime }d^{\prime }}$$

**Table 2.** Relative odds ratio from 2×2 tables by test.

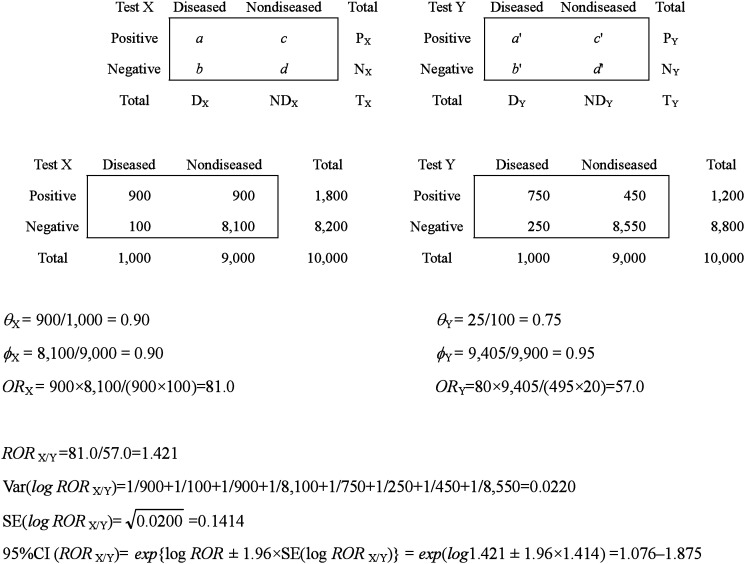

Because the variance of the *logOR* is calculated as (1/*a*)+(1/*b*)+(1/*c*)+(1/*d*), the variance of the difference between the *logOR*s is var(*logOR*_X_)+var(*logOR*_Y_) under the assumption of independence. Thus, we obtain the *ROR*, reflecting the relative diagnostic accuracy of test X to test Y, with a confidence interval (CI).

##### Indices based on 2×2 tables by disease status

Table 2 could be reconstructed to test results versus diagnostic tests by disease status as shown in Table 3. In this form, we can compare the sensitivities of the two tests as well as their specificities, applying the *χ*^2^ test for independence or a comparison of two proportions. These tests are mathematically equivalent.

**Table 3.** Relative odds ratio from 2×2 tables by disease status.

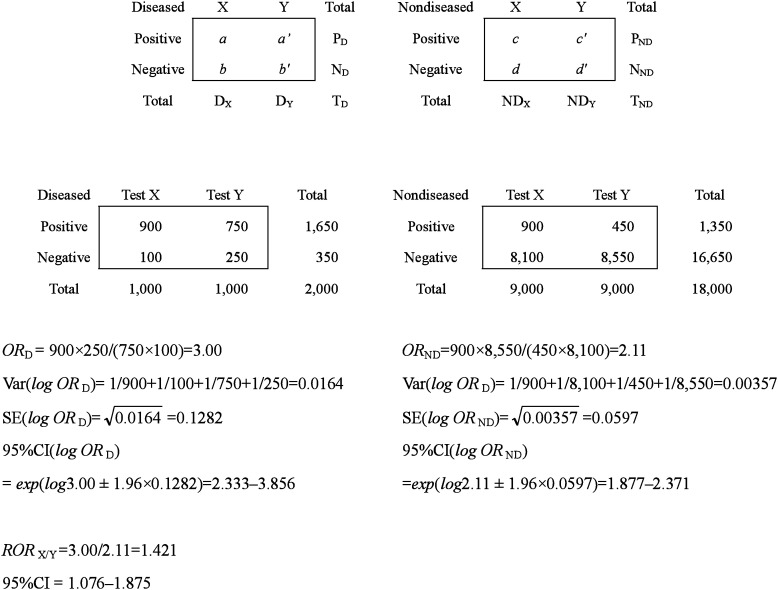

Another index for a comparison of the sensitivities and specificities of two tests is the *OR*, which is the ratio of the positive odds of test X to that of test Y in diseased or nondiseased subjects. As a positive result among the diseased subjects denotes a true positive, the *OR* among the diseased group (*OR*_D_= *ab*′/*a*′*b*) is the true positive odds ratio of test X against that of test Y, indicating the relative sensitivity of one to the other. CI of the *OR*_D_ is calculated using the variance of *logOR*_D_, which is (1/*a*)+(1/*b*)+(1/*a*′) +(1/*b*′) in Table 3. If *OR*_D_ is significantly greater than 1, the sensitivity of test X is higher than that of test Y. Similarly, a positive result in the nondiseased group is a false positive, and the *OR*_ND_ being *cd*′ /*c*′*d*, denotes a false positive odds ratio of test X to test Y. This index could also be used for the comparison of specificities. If *OR*_ND_ is smaller than 1, the specificity of test X is higher than that of test Y. The ratio of a true-positive to a false-positive odds ratio, *i.e.*, *adb*′*c*′/ *bca*′*d*′ is identical to the *ROR* calculated in Table 2. The variance and CI of the ratio are also identical to those in Table 2.

#### 2-2. Comparison of Diagnostic Accuracy of Two Tests Given to Same Individuals

When we compare the diagnostic accuracy of two tests given to different subjects, we should take into account the comparability of the subject groups to which each test was administered. Selection bias might invalidate the results on accuracy.^14^^-^^15^ Thus, we may give two diagnostic tests to the same individual, and try calculating the *ROR* in the same way. However, an *ROR* based on 2×2 tables by test requires the independence of both, which is not sufficient for a test with the same subjects. In that case, the *ROR* based on McNemar’s *OR* by disease status is available. As shown in Table 4, each number of the four cells in the ordinary 2×2 table (Table 3) moves to a marginal number in McNemar’s 2×2 table, and the result of test X with that of test Y of each individual is counted and classified into four cells. As McNemar’s table has more information than an ordinary 2×2 table, we can reconstruct the latter from the former, but not the other way around.

**Table 4.** McNemar’s 2×2 tables by disease status.

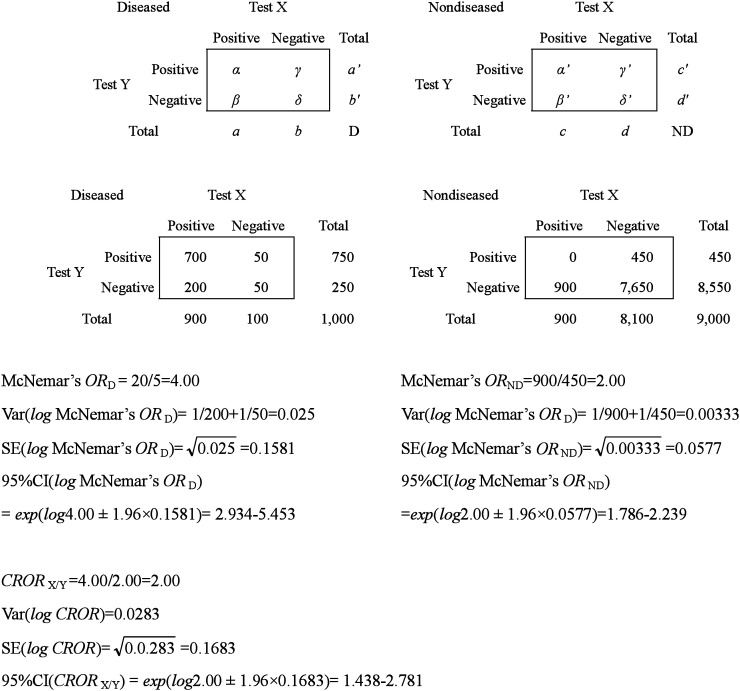

Because McNemar’s true-positive odds ratio is *β*/*γ*, and false-positive odds ratio is *β*′/*γ*′, the *ROR* using McNemar’s *OR*, the conditional relative odds ratio^16^ (*CROR*), is *βγ*′/*β*′*γ*. The CI of the newly proposed index is calculated from var(*logCROR*)=(1/*β*)+(1/*γ*)+(1/*β*′)+(1/*γ*′). The *CROR* requires the number of individuals having discordant results on the two tests, and no concordant results are needed.

### 3. Approach to Meta-Analysis of Comparison of Diagnostic Test Accuracy

There are two ways to compare diagnostic test accuracy using meta-analysis, *i.e.*, a comparison of two summary *OR*s of tests X and Y by extracting each *OR* from the original studies, and summarizing the *CROR* extracted from each. The SAS program for meta-analysis is provided elsewhere.^16^^-^^17^

#### Comparison of two summary ORs

Extracting the *OR* of each test from the original studies enables us to calculate summary *OR*s of tests X and Y with their variances. In order to summarize *OR*s, a proper model such as a fixed effect^18^^-^^19^ model or a random effect model^19^^-^^20^ can be used. A relative summary *OR* is calculated by dividing summary *OR*_X_ by summary *OR*_Y_. CI is computed using var(*log* relative summary *OR*)=var(*log* summary *OR*_X_)+var(*log* summary *OR*_Y_). This method is used when test X was given to different subjects than those who took test Y.

#### Summarizing CROR

We summarize the extracted *CROR* of test X to test Y using the same method as when summarizing the *OR*. This method is used when test X was given to the same individuals who took test Y.

## DISCUSSION

To evaluate diagnostic accuracy, *AR* is commonly used for its simplicity and ease of interpretation. However, a number of papers have reported its pitfalls.^4^^-^^6^^,^^21^ As *AR*, which is *pθ*+(1−*p*)*ϕ*, is the weighted mean of sensitivity and specificity, when prevalence is low, the sensitivity is almost neglected. In that case, *AR* does not convey the diagnostic accuracy of the test. An extreme example is shown in Table 5, showing a higher *AR* in test Y despite the fact that test Y has no diagnostic ability. In such a case, care should be taken to avoid accuracy comparison based on *AR*s of two groups.

**Table 5.** Comparison of two tests using several indices.

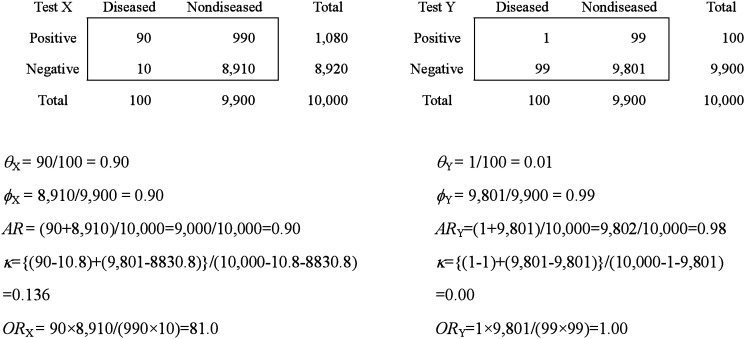

In spite of the improvement of *AR* with regard to removing chance agreement, attention should be paid to evaluating diagnostic accuracy using *κ*. As shown in Table 6, *κ* diminishes under fixed sensitivity and specificity when the prevalence is closer to one or zero. Even with the same prevalence, *κ* would be changed when the sensitivity and specificity are switched. These are examples of some undesirable features of *κ* for evaluating diagnostic accuracy.

**Table 6.** Agreement rate and kappa of diagnostic test under fixed odds ratio.

Sensitivity	Specificity	Prevalence	Odds ratio	Agreement rate	Kappa
0.9	0.8	0.01	36	0.801	0.0651
0.9	0.8	0.5	36	0.85	0.7
0.9	0.8	0.99	36	0.899	0.1206
0.8572	0.8572	0.01	36	0.8572	0.0901
0.8572	0.8572	0.5	36	0.8572	0.7144
0.8572	0.8572	0.99	36	0.8572	0.0901
0.8	0.9	0.01	36	0.899	0.1206
0.8	0.9	0.5	36	0.85	0.7
0.8	0.9	0.99	36	0.801	0.0651

The *OR* was originally used as an index representing the strength of a relationship between exposure and disease. It is essentially identical to the relationship between test results and the presence of disease. The remarkable feature of *OR* is its independence from prevalence and symmetry in terms of sensitivity and specificity. Moreover its variance is given by a simple formula. Therefore, the *OR* is widely used to evaluate and compare diagnostic accuracy, including use of the meta-analytic technique.^7^^-^^9^^,^^22^^-^^25^ However, as this index is based on odds, very small differences may sometimes be exaggerated. For example, a test with *θ*=0.9 and *ϕ*=0.9 has the same *OR* of 81 as another test with *θ*=0.99 and *ϕ*=0.45.

The *ROR* is used if two different tests to be compared are given to different subjects. Although the index is statistically correct, we should be careful of selection bias based on subject differences. To remove the bias, it would be preferable to give two tests to the same individuals. However, ordinal *ROR* assumes independence of two groups. In that case, the *CROR*, the ratio of a true-positive to a false-positive McNemar’s odds ratio, yields the correct answer to the question. The *CROR* is identical to the *ROR* when and only when each cell of McNemar’s 2×2 table is identical to the expected number from the margin. The *CROR* has the following characteristics: less biased index with CI considering the correlation of individual level of two tests, and economically profitable and ethically less problematic, because no diagnosis of disease is needed for subjects with negative results from both diagnostic tests. In addition, the *CROR* could be used in a comparison between the strength of association of two exposures to a disease.^26^ On the other hand, the *CROR* tends to have a broad CI because of the sparseness of McNemar’s tables. This phenomenon is quite serious when diagnostic tests are accurate, and consequently they are highly concordant. Finally, we should generally pay attention to the differences in the characteristics of two tests when we summarize sensitivity and specificity, being especially aware of any loss of information when summarizing indices.

In meta-analysis, although it is statistically appropriate to calculate the ratio of summary *OR*s by extracting the *OR* from each original study, selection bias would be generated if two tests were given to groups with different characteristics. Extracting ratio of the *OR* is more valid, because a comparison among the same subjects avoids selection bias. As long as the *CROR* is extracted (meaning that the *OR* is McNemar’s), there is no methodological problem. However, use of the ordinal *ROR* may be problematic, in particular a t-test of log*ROR* that ignores intra-study variations, which should be avoided since it leads to incorrectly low p-values.

The *CROR* is a new index, and at present can be extracted when raw data of discordant individuals are provided in an original study. In future studies of the comparative diagnostic accuracy of tests, the *CROR* should be presented if raw data of discordant individuals can not be presented for meta-analysis.

## Appendix

*-----Table 1&2-----;
data table1;
 do test='TestX', 'TestY' ; do disease=0 to 1; do result=0 to 1;
 input number @@; output; end; end; end;
 cards;
  8100 900 100 900 8550 450 250 750
  ;
 run;
proc format;
 value disfmt 1= 'Diseased' 0= 'Nondiseased' ;
 value pnfmt 1= '-Positive-' 0= ' =Negative=' ; run;
proc freq data=table1 order=formatted;
 tables test*result*disease /expected measures nocol norow nopct;
 format result pnfmt. disease disfmt.; weight number; output out=out rror;
 title 'Table 1&2' ; run;
data OR; set out;
 OR=_rror_; logOR=log(_rror_); SE=(log(u_rror/l_rror))/2/1.96;
 dummy=1;
 drop _rror_ u_rror l_rror; run;
proc sort; by test; run;
data table2; set OR; by dummy;
 retain OR0 SE0;
 drop test OR logOR SE dummy OR0 SE0 OR1 SE1 SEROR;
 if test= 'TestX' then do; OR0=OR; SE0=SE; end;
 if test= 'TestY' then do; OR1=OR; SE1=SE; end;
 SEROR=sqrt(SE0**2+SE1**2);
 ROR=OR0/OR1; LowROR=exp(log(ROR)-1.96*SEROR);
 HighROR=exp(log(ROR)+1.96*SEROR);
 if last.dummy then output;
proc print; title 'Table 2' ; run;
*-----Table 3-----;
proc freq data=table1 order=formatted;
 tables disease*result*test /measures nocol norow nopct;
 format result pnfmt. disease disfmt.; weight number; output out=out rror;
 title 'Table 3' ; run;
data OR; set out;
 OR=_rror_; logOR=log(_rror_); SE=(log(u_rror/l_rror))/2/1.96;
 dummy=1;
 drop _rror_ u_rror l_rror; run;
proc sort; by disease; run;
data table3; set OR; by dummy;
 retain OR0 SE0;
 drop disease OR logOR SE dummy OR0 SE0 OR1 SE1 SEROR;
 if disease=0 then do; OR0=OR; SE0=SE; end;
 if disease=1 then do; OR1=OR; SE1=SE; end;
 SEROR=sqrt(SE0**2+SE1**2);
 ROR=OR1/OR0; LowROR=exp(log(ROR)-1.96*SEROR);
 HighROR=exp(log(ROR)+1.96*SEROR);
 if last.dummy then output;
proc print; title 'Table 3' ; run;
*-----Table 4-----;
data table4;
 do disease=0 to 1; do X_Y= ' -P/N-' , '=N/P=' ;
 input number @@; output; end; end;
 cards;
  900 450 200 50
  ;
 run;

proc freq order=formatted;
 tables X_Y*disease/measures nocol norow nopct;
 format disease disfmt.; weight number;
 title 'Table 4 (See Odds Ratio)'; run;
*-----Table 5-----;
data table5;
 do test= 'TestX' , 'TestY' ; do disease=0 to 1; do result=0 to 1;
 input number @@; output; end; end; end;
 cards;
  8910 990 10 90 9801 99 99 1
  ;
 run;
proc freq order=formatted;
 tables result*disease /agree expected measures nocol norow nopct;
  format result pnfmt. disease disfmt.; weight number; by test;
  title 'Table 5' ; run;
*-----Table 6-----;
data table6;
 do test=1 to 9;
 input sens spec p @@;
 AR=p*sens+(1-p)*spec;
 kappa=2*p*(1-p)*(sens+spec-1)/(2*p*(1-p)*(sens+spec-1)+1-AR);
 OR=1/(1/sens-1)/(1/spec-1);
 output; end;
 cards;
  0.9 0.8 0.01 0.9 0.8 0.5 0.9 0.8 0.99
  0.8572 0.8572 0.01 0.8572 0.8572 0.5 0.8572 0.8572 0.99
  0.8 0.9 0.01 0.8 0.9 0.5 0.8 0.9 0.99
  ;
 run;
proc print; var test sens spec p OR AR kappa;
 title 'Table 6' ; run;
